# Efficacy and safety of Yirui capsule in patients with hyperlipidemia: study protocol for a multicenter, randomized, double-blind, placebo-controlled trial

**DOI:** 10.1186/s13063-016-1419-9

**Published:** 2016-06-18

**Authors:** Liang Dai, Linda L. D. Zhong, Yan Cao, Wei Chen, Ying Cheng, Xiu-Fang Lin, Zhao-Xiang Bian, Ai-Ping Lu

**Affiliations:** School of Chinese Medicine, Hong Kong Baptist University, 4/F, Jockey Club School of Chinese Medicine Building, 7 Baptist University Road, Kowloon Tong, Kowloon Hong Kong; Hong Kong Chinese Medicine Clinical Study Centre, Hong Kong Baptist University, 7 Baptist University Road, Kowloon Tong, Hong Kong, China; Health Management Center, the First Affiliated Hospital of Jinan University, No. 613 Huangpu Avenue West, Tianhe District Guangzhou, China; Outpatient Department, Guangdong No. 2 Provincial People’s Hospital, No. 466 Xingang Middle Road, Haizhu District Guangzhou, China; Department of Cardiology, Hexian Memorial Affiliated Hospital of Southern Medical University, No. 2 East Road of Qinghe, Shiqiao, Panyu District Guangzhou, China; Department of Cardiology, the Fifth Affiliated Hospital, Sun Yat-Sen University, No. 52 East Road of Meihua, Xiangzhou District Zhuhai, China

**Keywords:** Hyperlipidemia, Chinese medicine, Yirui capsules, Randomized controlled trial

## Abstract

**Background:**

Hyperlipidemia is a common disease in China. Although 88.9 % of the Chinese population taking lipid-lowering medications has already used a statin, only 61.5 % of the population has reached the goal of low-density lipoprotein cholesterol. Thus, many patients in China seek help from Traditional Chinese Medicine. Yirui capsules are an innovative Chinese Medicine which are designed to improve the blood lipid state in patients with hyperlipidemia. However, there is still a lack of high-quality evidence from clinical trials to support the application. Therefore, we designed a clinical trial to evaluate the safety and efficacy of Yirui capsules for use by patients with hyperlipidemia.

**Methods/design:**

This is a multicenter, randomized, double-blinded, placebo-controlled trial. Based on lifestyle modification therapy, eligible patients will randomly be assigned to the Yirui capsule or the placebo group. The primary outcome is the percentage of participants who reach the goal of 30 % low-density lipoprotein cholesterol decline at treatment end-point. The secondary outcomes include the changes from baseline to treatment endpoint in low-density lipoprotein cholesterol, total cholesterol, triglyceride, high-density lipoprotein cholesterol, apolipoprotein A, apolipoprotein B, non-high-density lipoprotein cholesterol, MOS 36-Item Short-Form Health Survey scoring, total and individual item scoring of symptomatic grading and quantifying scale, and body mass index.

**Discussion:**

The main ingredients of the Yirui capsule are perilla oil, *Folium Ginkgo* (Yinxingye), *Radix Salviae miltiorrhizae* (Danshen), *Fructus Crataegi* (Shanzha), *Rhizoma Alismatis* (Zexie), and *Radix Notoginseng* (Sanqi), which are expected to improve the blood lipid state. This randomized placebo-controlled trial will comprehensively evaluate the effectiveness and safety of Yirui capsules against hyperlipidemia in the hope of providing a new adjunctive Chinese medicine option for clinical practice in dyslipidemia treatment.

**Trial registration:**

ChiCTR-IOR-15006496. Registered on 29 May 2015.

**Protocol version:**

WXJ.YRJN-HBT-V1.0 (21 Jan 2015).

## Background

Due to the unhealthy lifestyle of modern society, hyperlipidemia has become a common disease in China. According to the latest epidemiological research, more than 300 million adults in the Chinese population aged ≥20 years have a total cholesterol (TC) level ≥5.18 mmol/L and approximately 200 million people have a low-density lipoprotein cholesterol (LDL-C) level ≥3.37 mmol/L [1]. Hyperlipidemia is considered to be one of the major risk factors for the development of atherosclerosis and coronary heart disease (CHD) [2–4]. A recent meta-analysis demonstrated that a 1 mmol/L LDL-C decrease could result in a 21 % reduction in risk for a major vascular event [5]. Therefore, lipid-lowering therapy has significant value in preventing cardiovascular disease.

Statins are the most common drug used in lipid-lowering therapy. Of the Chinese population using lipid-lowering medications, 88.9 % of them used statins. However, only 61.5 % of the population reached the goal of decreased LDL-C [6]. There may be various reasons for this unsatisfied goal attainment. For instance, utilization of moderate/high-intensity statins is a common method for achieving the LDL-C goal. However, increasing the intensity of statins may lead to a high risk of adverse events, which results in low compliance and thus unsatisfactory goal attainment [7–9]. Moreover, in China, there is only one category of statin, simvastatin, which happens to be a low-intensity statin, on the National Essential Drugs List [10]. The high cost of other moderate/high-intensity statins may also limit their usage. Therefore, to help reach their goal attainment, many patients in China will seek help from Traditional Chinese Medicine (TCM).

Based on TCM theory, the generation of hyperlipidemia has a close relationship with the liver, spleen, kidney, and diet. The basic pathogenesis is phlegm-dampness stagnation and blood vessel obstruction by phlegm and blood stasis. Multiple reasons such as a deficiency of the spleen, over intake of greasy foods, and stagnation of the liver qi could lead to this pathological state. Therefore, according to the therapeutic principle of promoting blood circulation to remove blood stasis and promoting diuresis to eliminate dampness, the Yirui capsule is an innovative Chinese Medicine (CM) which could help hyperlipidemia patients to improve their blood lipid state.

The major contents of Yirui capsule include perilla oil, *Folium Ginkgo* (Yinxingye), *Radix Salviae miltiorrhizae* (Danshen), *Fructus Crataegi* (Shanzha), *Rhizoma Alismatis* (Zexie), and *Radix Notoginseng* (Sanqi). A modern pharmacological study showed that the main functional component of Yirui capsules was alpha-linolenic acid [11]. Recent studies have illustrated that alpha-linolenic acid could improve vascular inflammation and endothelial function and be helpful in secondary prevention of CHD [12–14]. The lipid-lowering effect of other herbal components has also been illustrated in animal experiments [15–19]. Possible mechanisms may relate to limitation of absorption, promotion of the metabolism, and transformation of blood lipids. As a Chinese Herbal Medicine, Yirui capsules could help prevent the generation of hyperlipidemia and regulate the blood lipid level. Two animal experiments have been completed and demonstrated that Yirui capsules could significantly reduce the LDL-C level in hyperlipidemic rat models [20, 21]. Nevertheless, there is still a lack of high-quality evidence from clinical trials to support the application. In the current study, we aim to demonstrate and assess the efficacy and safety of Yirui capsules in improving hyperlipidemia by using a randomized controlled trial (RCT). We chose simulant Yirui capsules which contained only a placebo as a comparator so that the actual lipid-lowering effect of the Yirui capsules could be evaluated.

The study was financially supported by Infinitus (China) Company Limited and was registered with an identifier (ChiCTR-IOR-15006496, 29 May 2015) in the Chinese Clinical Trial Registry (ChiCTR). This funding source had no role in the design of this study and will not have any responsibility during its execution, analyses, interpretation of the data, or decision to submit results.

## Methods/design

### Study design

**Fig. 1:**
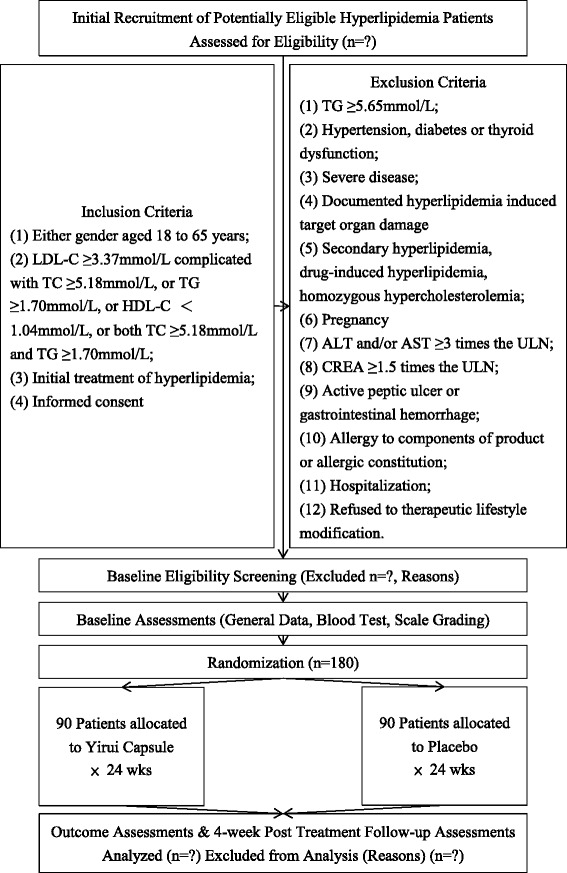
Participant flowchart

This study is conducted as a double-blind, placebo-controlled trial with two parallel groups to examine the blood lipid-lowering effect of Yirui capsules. The protocol was developed according to Consolidated Standards of Reporting Trials (CONSORT) statement [22], Standard Protocol Items: Recommendations for Interventional Trials (SPIRIT) 2013 [23], and SPIRIT 2013 explanation and elaboration: guidance for protocols of clinical trials [24]. We plan to recruit 180 patients from the First Affiliated Hospital of Jinan University, Guangdong No. 2 Provincial People’s Hospital, Hexian Memorial Affiliated Hospital of Southern Medical University, and the Fifth Affiliated Hospital, Sun Yat-sen University. Clinical investigators from each center are responsible for screening eligible participants. After recruitment, participants are randomly and equally divided into two groups, the Yirui capsule group and the placebo group. Both arms will receive lifestyle modification therapy (decreasing the intake of saturated fatty acids and cholesterol, selecting foods which could reduce LDL-C, losing weight, increasing regular physical exercise, quitting smoking, limiting salt intake, etc.). Based on lifestyle modification, the Yirui group will receive Yirui capsules while the placebo group will receive simulant capsules. Then, the two arms will undergo a 24-week treatment and 4-week follow-up period. According to the China Food and Drug Administration guidelines [25], this 24-week treatment period was set based on our clinical experience and previous pilot studies. The participant flowchart is shown in Fig. 1, and the participant timeline is given in Table 1.

**Table 1:**
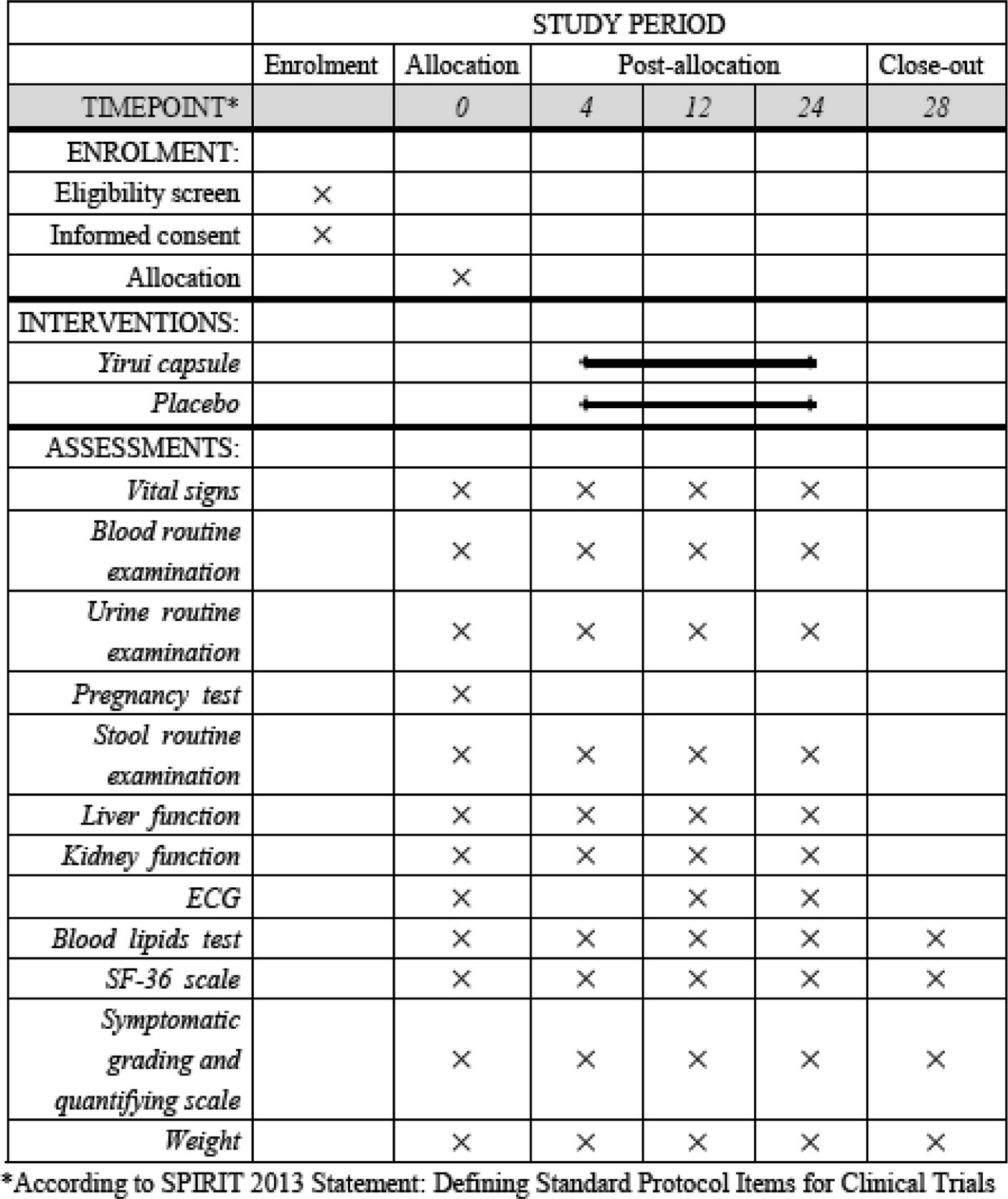
Schedule of enrollment, intervention, and assessments

There will be five visits for each participant in week 0, week 4, week 12, week 24, and week 28. At the first visit, patients will have their general data recorded and will be given the prescription. Then, patients will take the prescription to the specified pharmacy and receive either Yirui capsules or the placebo. 4 weeks and 12 weeks after the first visit, patients will come back for clinical evaluation and medication. Visit 4 is planned for the end of the treatment period (week 24); at this visit patients will only receive clinical evaluation. Afterwards, the patients will stop taking Yirui capsules or the placebo for another 4 weeks and then come back for clinical evaluation.

The study protocol was approved by the ethics committees of the First Affiliated Hospital of Jinan University, Guangdong No. 2 Provincial People’s Hospital, Hexian Memorial Affiliated Hospital of Southern Medical University, and the Fifth Affiliated Hospital, Sun Yat-sen University (Approval Version: LWXJ.YRJN-HBT-V1.0), and informed consent was obtained from each participant.

### Participants

#### Setting

The trial is conducted at the First Affiliated Hospital of Jinan University, Guangdong No. 2 Provincial People’s Hospital, Hexian Memorial Affiliated Hospital of Southern Medical University, and the Fifth Affiliated Hospital, Sun Yat-sen University. All participants are recruited from the public through the outpatient clinic.

#### Inclusion criteria

In this study, the inclusion criteria are as follows: (1) age of 18 to 65 years, both genders; (2) documented blood lipid test results indicated LDL-C ≥3.37 mmol/L complicated with TC ≥5.18 mmol/L, or triglyceride (TG) ≥1.70 mmol/L, or high-density lipoprotein cholesterol (HDL-C) <1.04 mmol/L, or both TC ≥5.18 mmol/L and TG ≥1.70 mmol/L; (3) initial treatment of hyperlipidemia; (4) signed informed consent and agreement to complete all visits, examinations, and treatments.

#### Exclusion criteria

Patients were excluded for one or more of the following reasons: (1) TG ≥5.65 mmol/L; (2) combined with hypertension, diabetes, or thyroid dysfunction; (3) combined severe primary diseases (i.e., cardiovascular and cerebrovascular diseases, liver and kidney diseases, hematopoietic system dysfunction), or malignant tumor, or mental disorder, or other serious complicated disease; (4) documented hyperlipidemia-induced target organ damage (i.e., atherosclerosis and CHD) with need to maintain treatment to dyslipidemia; (5) secondary hyperlipidemia, drug-induced hyperlipidemia, homozygous hypercholesterolemia; (6) pregnancy; (7) alanine aminotransferase (ALT) and/or aspartate aminotransferase (AST) ≥3 times the upper limit of normal (ULN); (8) creatinine (CREA) ≥1.5 times the ULN; (9) combined with active peptic ulcer or gastrointestinal hemorrhage; (10) allergy to components of product or allergic constitution; (11) hospitalized; (12) cannot obey therapeutic lifestyle modification.

### Interventions

#### Lifestyle modification

As mentioned above, every participant should practice therapeutic lifestyle modification. Specifically, the recommendations include reducing the intake of saturated fatty acids and cholesterol, choosing foods which could reduce LDL-C, losing weight, increasing regular physical exercise, smoking cessation, and limiting salt intake, etc. Diet evaluation should be conducted every week by the participant him/herself and the information documented in a diet diary card. The specific items are listed in Table 2.

**Table 2:** Diet evaluation diary card

Items	Scoring
1. Did you eat meat <75 g/day in one recent week? 0 = No, 1 = Yes	
2. What kinds of meat did you eat: 0 = lean meat, 1 = fat lean meat, 2 = fat meat, 3 = innards	
3. How many eggs did you eat in one recent week? 1 = 0–3 eggs, 2 = 4–7 eggs, 3 = more than 7 eggs	
4. How many times did you eat fried food in one recent week? 0 = Not eaten, 1 = 1–4 times, 2 = 5–7 times, 3 = more than 7 times	
5. How many times did you eat brioches in one recent week? 0 = Not eaten, 1 = 1–4 times, 2 = 5–7 times	
Total	

#### Chinese Medicine intervention

The Yirui capsule is composed of perilla oil, *Folium Ginkgo* (Yinxingye), *Radix Salviae miltiorrhizae* (Danshen), *Fructus Crataegi* (Shanzha), *Rhizoma Alismatis* (Zexie), and *Radix Notoginseng* (Sanqi). The specific Chinese herbal medicine composition and actions are summarized in Table 3. The placebo capsule was a simulant Yirui capsule with a comparable appearance. The main content of the placebo capsule was starch. By adding food colorants and flavoring agents, the mixture achieved a color, smell, taste, and texture comparable to the content of the Yirui capsule. Participants from both the Yirui group and the placebo group were informed to take two assigned capsules orally twice daily for 24 weeks continuously. Both Yirui capsules and the placebo were packed in sealed plastic bottles (120 capsules each). Only the serial number was printed outside the package to ensure successful blinding of patients.

**Table 3:** Composition and action of Chinese herbal medicine in Yirui capsule

Ingredients	Action
*Folium Ginkgo*	TCM: Promoting blood circulation to remove blood stasis and stop pain Pharmaceutical study: (1) cardiovascular protective effect, (2) hypolipidemic effect, (3) effect of antagonizing lipid peroxide, (4) effect of improving cerebral circulation
*Radix Salviae miltiorrhizae*	TCM: Promoting blood circulation to remove blood stasis, cooling blood and treating boils, tranquilizing mind Pharmaceutical study: (1) cardiovascular protective effect, (2) hypotensive effect, (3) hypolipidemic effect, (4) anti-inflammatory effect, (5) anti-coagulation effect
*Fructus Crataegi*	TCM: Removing food retention and flatulence, promoting blood circulation to remove blood stasis Pharmaceutical study: (1) gastrointestinal regulation effect, (2) cardiovascular protective effect, (3) hypotensive effect, (4) hypolipidemic effect
*Rhizoma Alismatis*	TCM: Eliminating dampness and diuresis, purging heat Pharmaceutical study: (1) diuretic effect, (2) hypolipidemic effect, (3) anti-allergic effect, (4) anti-inflammatory effect
*Radix Notoginseng*	TCM: Hemostatic elimination of stagnation, promoting blood circulation to stop pain Pharmaceutical study: (1) Anti-coagulation effect, (2) hematopoiesis effect, (3) cardiovascular protective effect, (4) analgesic effect, (5) anti-inflammatory effect

### Outcomes

Clinical investigators will obtain written informed consent and collect basic data such as gender, age, height, weight, and medical history at baseline. For safety concerns, besides the basic data, vital signs will be taken and laboratory examinations conducted, for instance, blood pressure, heart rate, routine blood tests (e.g., red blood cell (RBC), white blood cell (WBC), hemoglobin (Hb), etc.), liver and kidney function tests (e.g., ALT, AST, CREA, etc.), pregnancy test, and 12-lead electrocardiogram (ECG). Except for the pregnancy test, which only needs to be conducted in the first visit (week 0, baseline), other tests should be conducted routinely at every visit during the treatment period (the ECG is not mandatory at the second visit (week 4)). Also, any adverse events such as abdominal discomfort should be monitored and recorded during the whole study.

Participants need to receive a blood lipid profile test which contains the following indexes: TC, TG, HDL-C, LDL-C, apolipoprotein A (apoA), apolipoprotein B (apoB), and non-high-density lipoprotein cholesterol (non-HDL-C). The blood sample should be collected after at least a 12-hour fast, and the last meal before the blood sampling should not contain greasy food. In order to evaluate the health status of participants, the MOS 36-Item Short-Form Health Survey (SF-36) is utilized [26–28]. In addition, for assessment of clinical symptoms, another symptomatic grading and quantifying scale is applied. Weight monitoring and diet evaluation are also two aspects that need to be conducted. All participants are required to complete the tests and scales mentioned above at every visit under the guidance and assistance of the clinical investigators. The results are recorded and compared within a group and between groups before and after intervention.

Yirui capsules are designed to act as an intervention method for patients with borderline high serum lipid or an adjunctive choice for patients who could not reach the serum lipid target with conventional therapy. Based on the 2013 American College of Cardiology (ACC)/American Heart Association (AHA) guidelines on the treatment of blood cholesterol [29], the 2007 Chinese guidelines on blood lipid abnormality [30], and a previous TCM clinical trial [31], we set the treatment goal as a 30 % LDL-C decline compared with baseline after intervention. Therefore, the primary outcome is the percentage of participants who reach the goal at treatment endpoint (week 24). Secondary outcomes include the mean changes from baseline at treatment endpoint in LDL-C, TC, TG, HDL-C, apoA, apoB, non-HDL-C, SF-36 scoring, total and individual item scoring of symptomatic grading and quantifying scale, and body mass index (BMI).

Safety and tolerability assessments include collection of adverse events, physical examination, vital signs, and the relevant laboratory examination reported previously. Clinical investigators should observe any adverse events that occur during the clinical trial, including abnormal symptoms and signs, unusual laboratory indicators, etc. Whether or not the adverse event is related to the interventions, investigators are required to record it comprehensively, including onset time, related symptoms and signs, extent, duration, laboratory indexes, treatment, result, and follow-up. In addition, drug combinations should also be documented in order to analyze the correlation between adverse events and experimental products.

### Assignment and blinding

Block randomization is conducted in a 1:1 ratio to receive either Yirui capsules or the placebo. As this study is a multicenter study, blocked randomization is conducted for each center. The randomization sequence table generated by Statistical Analysis System (SAS) Version 9.2 was provided by a specific statistical researcher who does not participate in the clinical trial. The statistical researcher randomly distributes the number to the research products. The random numbers are kept in opaque sealed envelopes and sent to research centers. Afterwards, clinical investigators randomly assign the random number based on enrollment order and provide the corresponding drug. Choosing the number intentionally is strictly forbidden. The random number is fixed during the whole study. The blinding base containing the randomization sequence, parameters of sequence, and random number is sealed in duplicate and reserved by the principal investigator and product registration applicant. Patients and clinical investigators can only be aware of the individual random number. Treatment assignments are not exposed and are blinded to patients and any clinical investigators involved in this trial until the entire study is completed. At the last visit, all participants and clinical investigators will finish a questionnaire about which treatment (Yirui capsule group or placebo group) the patients received to assess the success of blinding. Emergency letters containing random number and treatment assignment would also be prepared by a specific statistical researcher. Code breaks are only allowed in emergency situations when practical intervention is compulsory for further management of the participant.

### Sample size calculation

The sample size calculation was based on the primary outcome measure. Previous studies of Chinese herbal medicine (CHM) on hyperlipidemia had detected a difference of at least 20 % between Yirui capsule and placebo. Assume that placebo effect is about 10 %. The sample size in each group was determined by using the formula derived by Whitley et al. [32]:

$$ n=\frac{\left[{\mathrm{p}}_1\left(1-{\mathrm{p}}_1\right)+{\mathrm{p}}_2\left(1-{\mathrm{p}}_2\right)\right]}{{\left({\mathrm{p}}_1-{\mathrm{p}}_2\right)}^2}\times {C}_{\mathrm{p},\kern0.5em \mathrm{power}} $$

For type I: α = 0.05, β = 0.10; power (1-β) = 90 %; p_1_ = 10 %, p_2_ = 30 %; and C _p, power_ = 10.5; the two-tailed level α and power were set at 0.05 and 80 %, respectively. Seventy-five subjects were needed in each group. Estimating about 20 % dropouts, the number of each group was 90, and a total of 180 subjects were needed for this study.

### Statistical analysis

All effectiveness and safety evaluations will be conducted according to the intention-to-treat (ITT) principle. The last-observation-carried-forward method will be applied to deal with missing values. SAS (Version 9.2) will be utilized to analyze the data, and the statistical significance is defined as a two-sided *P* value of <0.05. For measurement data, the mean, standard deviation (SD), maximum, minimum, P25, median, and P75 will be reported. For paired measurement data, the mean difference, SD, and confidence interval (CI) will also be reported. As to enumeration data and ranked data, frequency distribution and corresponding percentage are compulsory. Baseline data of the two arms (e.g., demographic data) will undergo descriptive and inferential analyses. Intra-group efficacy comparisons before and after the treatment will be conducted by using the paired *t* test or Wilcoxon signed rank test for quantitative variables and the chi-squared test for qualitative variables. Efficacy difference between groups will be assessed with the utilization of the two-sample *t* test or corresponding non-parameter methods for quantitative variables, the chi-squared test for qualitative variables, and the Wilcoxon rank sum test for rank variables. For the primary outcome, the mean difference with two-sided 95 % CI will be reported. Statistic effectiveness is defined as the lower limit of 95 % CI above 0. Clinical effectiveness needs to be assessed after combining with clinical significance. For the consideration of the multicenter character, a covariance model is used to evaluate the center effect for the primary variable. Other secondary outcomes will take no account of the center effect. In addition, the chi-squared test will be utilized to analyze the rate of adverse events between the two arms. A table will be generated to enumerate and describe the characters of all adverse events during the study.

### Data collection and handling of withdrawal and dropout

In this 28-week clinical trial, it is compulsory for participants to take experimental medication for a continuous 24 weeks, be present at five assessment visits, conduct several blood tests, finish certain scales, complete a diet evaluation diary card, and stop taking other lipid-lowering medication and drugs with relevant herbal ingredients. Specially assigned individuals will record the original data, and the relevant materials will be placed on file based on numerical order at the specified site appropriately. Only principal investigators and clinical investigators have the right to examine the documents. Original documents should be preserved at least 5 years after finishing the study.

For maximum compliance of patients, we require all researchers and doctors to obey the following rules. Firstly, all researchers should obey the principle of informed consent and help participants understand the requirements of the study. Secondly, researchers need to monitor the compliance of patients through the dose counting method. Thirdly, all doctors should ask patients to bring all medications to every visit to examine and record any drug combinations. Moreover, investigators will contact the participant to remind them of the visit three days in advance and to inform them of the specific arrangements for the next visit. For patients who do not respond to the medication or cannot take medication on time, increasing the frequency of the follow-up visits is compulsory.

Participants can quit the study if they experience: (1) severe adverse effect(s), (2) hypersensitivity towards the research medication, or (3) aggravation of disease. The whole research plan will be terminated for the following circumstances: (1) presence of serious adverse events related to the research medication, (2) no practical value findings of the medication during the treatment, or (3) completion of all follow-up assessments.

## Discussion

Hyperlipidemia is now becoming a frequent illness in clinical practice. An overwhelming majority of patients choose statins as the first option of lipid-lowering therapy. Recently, considering the fact that the LDL-C theory of “lower is better” has been illustrated repeatedly [5, 33, 34], the latest guidelines point out that lipid-lowering therapy should select high- or moderate-intensity statins [2]. However, high-intensity statins also bring a high risk of adverse events [35]. The study of Preiss et al. even found that intensive statin therapy would increase the risk of new onset diabetes compared with moderate statin therapy [36]. Moreover, for the Chinese population, it has been reported that the pharmacokinetic characteristics of rosuvastatin in Asian people are different than in Caucasians, which may lead to the drug accumulation and increase the risk of adverse events [37]. Therefore, ways of attaining the LDL-C goal with relatively low-intensity statins combined with other kinds of drugs has become a popular research topic.

TCM has been used for thousands of years to treat diseases. Many classic formulas have been modified by modern techniques and applied in clinical practice. The Yirui capsule is a kind of CM used to regulate blood circulation. The main ingredients are perilla oil, *Folium Ginkgo* (Yinxingye), *Radix Salviae miltiorrhizae* (Danshen), *Fructus Crataegi* (Shanzha), *Rhizoma Alismatis* (Zexie), and *Radix Notoginseng* (Sanqi). In TCM theory, these components have the effect of promoting blood circulation and dispelling dampness; blood stasis and dampness provide the crucial pathogenesis of hyperlipidemia in the TCM concept. Moreover, a recent study proved that perilla oil was effective in improving the blood lipid profile of patients with hyperlipidemia [38]. The lipid-lowering effect of other Chinese Herbal Medicine ingredients was also demonstrated in animal experiments [15–19]. Therefore, by conducting this randomized placebo-controlled trial, we hope to verify the lipid-lowering effect of Yirui capsules and thus provide a new adjunctive option for clinical practice.

The clinical trial protocol is the base of the whole study. It plays a crucial role through study preparing, proceeding, reporting, and evaluation. This protocol has been developed according to CONSORT statement [21], SPIRIT 2013 [22], and the SPIRIT 2013 explanation and elaboration: guidance for protocols of clinical trials [23] in order to establish an appropriate standard for scientific, ethical, and safety issues of the study.

In conclusion, the results of the study are expected to provide exact efficacy and safety evidence of Yirui capsules in treating hyperlipidemia. This may help provide clinicians with another option for adjunctive lipid-lowering treatments based on statins.

## Trial status

At the time of manuscript submission, patients are being recruited, and no one has yet completed the treatment.

## Abbreviations

ALT, alanine aminotransferase; apoA, apolipoprotein A; apoB, apolipoprotein B; AST, aspartate aminotransferase; BMI, body mass index; CHD, coronary heart disease; CI, confidence interval; CM, Chinese medicine; CONSORT, Consolidated Standards of Reporting Trials; CREA, creatinine; ECG, electrocardiogram; Hb, hemoglobin; HDL-C, high-density lipoprotein cholesterol; ITT, intention-to-treat; LDL-C, low-density lipoprotein cholesterol; non-HDL-C, non-high-density lipoprotein cholesterol; RBC, red blood cell; RCT, randomized controlled trial; SAS, Statistical Analysis System; SD, standard deviation; SF-36, MOS 36-Item Short-Form Health Survey; SPIRIT, Standard Protocol Items: Recommendations for Interventional Trials; TC, total cholesterol; TCM, traditional Chinese medicine; TG, triglyceride; ULN, upper limit of normal; WBC, white blood cell
